# Reconstituted Human Upper Airway Epithelium as 3-D *In Vitro* Model for Nasal Polyposis

**DOI:** 10.1371/journal.pone.0100537

**Published:** 2014-06-19

**Authors:** Francisco de Borja Callejas, Asunción Martínez-Antón, Isam Alobid, Mireya Fuentes, Julio Cortijo, César Picado, Jordi Roca-Ferrer, Joaquim Mullol

**Affiliations:** 1 Clinical and Experimental Respiratory Immunoallergy, Institut d’Investigacions Biomèdiques August Pi i Sunyer (IDIBAPS), Barcelona, Spain; 2 CIBER of Respiratory Diseases (CIBERES), Barcelona, Spain; 3 Rhinology Unit & Smell Clinic, ENT Department, Hospital Clínic, Universitat de Barcelona, Barcelona, Catalonia, Spain; 4 Pneumology & Respiratory Allergy Department, Hospital Clínic, Universitat de Barcelona, Barcelona, Catalonia, Spain; Comprehensive Pneumology Center, Germany

## Abstract

**Background:**

Primary human airway epithelial cells cultured in an air-liquid interface (ALI) develop a well-differentiated epithelium. However, neither characterization of mucociliar differentiation overtime nor the inflammatory function of reconstituted nasal polyp (NP) epithelia have been described.

**Objectives:**

1^st^) To develop and characterize the mucociliar differentiation overtime of human epithelial cells of chronic rhinosinusitis with nasal polyps (CRSwNP) in ALI culture system; 2^nd^) To corroborate that 3D *in vitro* model of NP reconstituted epithelium maintains, compared to control nasal mucosa (NM), an inflammatory function.

**Methods:**

Epithelial cells were obtained from 9 NP and 7 control NM, and differentiated in ALI culture for 28 days. Mucociliary differentiation was characterized at different times (0, 7, 14, 21, and 28 days) using ultrastructure analysis by electron microscopy; ΔNp63 (basal stem/progenitor cell), β-tubulin IV (cilia), and MUC5AC (goblet cell) expression by immunocytochemistry; and mucous (MUC5AC, MUC5B) and serous (Lactoferrin) secretion by ELISA. Inflammatory function of ALI cultures (at days 0, 14, and 28) through cytokine (IL-8, IL-1β, IL-6, IL-10, TNF-α, and IL-12p70) and chemokine (RANTES, MIG, MCP-1, IP-10, eotaxin-1, and GM-CSF) production was analysed by CBA (Cytometric Bead Array).

**Results:**

In both NP and control NM ALI cultures, pseudostratified epithelium with ciliated, mucus-secreting, and basal cells were observed by electron microscopy at days 14 and 28. Displaying epithelial cell re-differentation, β-tubulin IV and MUC5AC positive cells increased, while ΔNp63 positive cells decreased overtime. No significant differences were found overtime in MUC5AC, MUC5B, and lactoferrin secretions between both ALI cultures. IL-8 and GM-CSF were significantly increased in NP compared to control NM regenerated epithelia.

**Conclusion:**

Reconstituted epithelia from human NP epithelial cells cultured in ALI system provides a *3D in vitro* model that could be useful both for studying the role of epithelium in CRSwNP while developing new therapeutic strategies, including cell therapy, for CRSwNP.

## Introduction

The mucosa of the nose and paranasal sinuses is lined by a pseudostratified epithelium, formed by ciliated and non-ciliated columnar cells, goblet cells and basal cells [1], [2]. This epithelium plays a crucial role in maintaining the homeostasis of both nasal and sinonasal mucosa. It is the first line of host defense against inhaled pollutants, allergens, and microbial pathogens [3], [4], it regulates not only innate but also acquired immunity through production of a wide range of cytokines, chemokines, and mediators [5], [6], and it is also able to repair and remodel its structure and integrity after epithelial damage [2], [7]. However, when nasal and sinonasal mucosa are chronically inflamed, such as in chronic rhinosinusitis with nasal polyps (CRSwNP), the epithelium function and structure become altered [1], [2].

CRSwNP is a disease of unknown etiology, characterized by a persistent symptomatic inflammation of the nasal and sinonasal mucosa [1]. In patients with CRSwNP, the epithelium is damaged (partial shedding, complete denudation, or loss of cilia) and shows an abnormal remodeling (goblet cell hyperplasia, basal cell hyperplasia, or metaplasia) [2], [8]. As a consequence, the identification of molecular mechanisms of the upper airway epithelial cells involved in repair, proliferation, and mucociliary differentiation under normal and pathological conditions, offers some potential for the development of new strategies for CRSwNP treatment.

A number of *in vitro* methods have been previously developed to investigate the nasal epithelium biology and physiopathology in human airways [9]–[11]. The human nasal RPMI-2650 cell line, derived from squamous cell carcinoma of nasal septum, are widely used as an *in vitro* cell culture system due to are easily maintained in culture, has extended lifespan, improved proliferation and homogeneity [12], [13], but they do not have the morphology, biochemical characteristics, and cellular response of control nasal epithelial cells [11]. On the other hand, primary cells cultured in submerged culture, clearly undergo a dedifferentiation and loss of the original *in vivo* phenotype [9], [14]. An ideal human nasal epithelium *in vitro* model would require a morphologically well differentiated culture, with ciliated, non-ciliated, secretory, and basal cells, while showing epithelial function (barrier formation, mucus secretion, ciliary activity, cytokines and chemokines signalling) [10], [11]. These requirements are only present in the following *in vitro* culture systems: organotypic explant culture or primary cells cultured at the air-liquid interface (ALI). The former maintain the original epithelium whereas the latter mimic the epithelium. Organotypic explants are *ex vivo* models of nasal mucosa that can be cultured maintaining intact the original epithelium. In fact, they have been widely used to study the human normal [15], [16], [17] and diseased [18] nasal mucosa. However, due to presence of numerous cell types, matrices, and other environmental factors, explant culture models are less homogeneous, standarized, and reproducible than ALI culture models for primary epithelial cells (Table 1). It is well-known that the ALI system provides a well differentiated culture that has been developed in both human upper [19]–[23] and lower [19], [24]–[29] airways, as well as in different animals [30]–[32]. Human nasal epithelial cells cultured in an ALI system represent the most promising experimental tool for investigating repair and differentiation, as well as to perform pharmacological, toxicological, and transport studies [11], [25], [28], [33].

**Table 1:** Comparison between 3D *in vitro* model of reconstituted epithelium and organotypic explant culture.

	*Straight Tissue* *Culture/Sections* *of Tissue*	*3D In* *Vitro* *Reconstituted Epithelium*
**Maintain Original Epithelium**	**Yes**	**No**
		**(but similar)**
**Different Tissue** **Structures and/or Cells**	**Yes**	**No**
(Heterogeneity)	**(tissue section)**	
**Infiltrated Inflammatory Cells**	**Yes**	**No**
**Controlled Environment**	**Yes**	**Yes**
**Experimental Studies** **within Epithelium**	**Yes**	**Yes**
(with pro-inflammatory,anti-inflammatory, and othermediators, mucociliar differentiation…)	**(non-specific)**	
**Test Drugs in Epithelium**	**Yes**	**Yes**
(Effects, Toxicity, and theirTransepithelial Transport)	**(non-specific)**	
**Functional Genetic Studies**	**No**	**Yes**
(Gene/small RNAsTransfection/Transduction,Gene Silencing…)		
**Study of Mucous and** **Serous Secretions** **from the Epithelium**	**Yes**	**Yes**
(basal, induced, or inhibited)	**(but altered by glands)**	
**Study of Cytokine** **and Chemokine** **Secretion from** **the Epithelium**	**Yes**	**Yes**
(basal, induced, or inhibited)	**(non-specific due** **to inflammatory and** **other cells)**	

Mucociliary differentiation in ALI culture models has been described with human nasal epithelial cells from inferior turbinates [19], [22], [23], and nasal polyps (NP) [20], [21]. Concerning to NP, studies from Bleier et al. [20] and Hajj et al. [21] described that it is possible to regenerate NP epithelium by culturing isolated epithelial or adult basal cells from NP at the ALI culture system, respectively. The former study demonstrated that NP reconstituted epithelium retained their primary phenotype with respect to ciliary function and epithelial permeability, whereas the latter study provided evidence that restored epithelium was differentiated and showing intact immune barrier functions by secreting factors that kill bacteria. However, neither characterization of mucociliar differentiation over time of NP epithelial cells in ALI cultures, nor the inflammatory function of NP reconstituted epithelia, have been described.

Therefore, the aims of this study were to develop and characterize the mucociliary differentiation over time of NP epithelial cells cultured at the ALI culture, and to validate the inflammatory function of the *in vitro* NP reconstituted epithelia by measuring their cytokine and chemokine secretions, in comparison to control nasal mucosa (NM).

## Materials and Methods

### Ethics Statement

All patients signed informed consent to participate in the study, which was approved by our institution’s Scientific and Ethics Committee:

- Name of approval committee: Ethics Committee for Clinical Research, Hospital Clínic, University of Barcelona.
- Date on which approval was given: 12/04/2012.
- Document number of approval: 2012\7466.

### Study Population

Nasal polyp specimens (N = 9) were obtained from patients undergoing endoscopic sinus surgery for severe CRSwNP, a negative skin prick test to common allergens, and without bronchial asthma or aspirin intolerance. The clinical diagnosis of CRSwNP was based on nasal symptoms, presence of nasal polyp by nasal endoscopy, and bilateral sinus opacification detection by computed tomography scan, following EPOS criteria [1]. Patients with cystic fibrosis were excluded from the study.

Control nasal mucosa specimens (n = 7) were obtained from inferior turbinates of subjects undergoing septoplasty or partial turbinectomy. All subjects with negative skin prick test to common allergens, without bronchial asthma or aspirin intolerance.

None of the patients and subjects involved in the study had had an upper respiratory tract infection within 2 weeks prior to surgery. Neither were they undergoing topical or systemic corticosteroid treatment during the 4 weeks leading up to surgery.

### Tissue Handling and Epithelial Cell Isolation from the Explant Culture

Tissue specimens were surgically removed and immediately transported to laboratory in sterile growth medium composed of BEBM (Bronchial Epithelial Cell Basal Medium; Clonetics, Lonza Group Ltd, Switzerland) supplemented with Epidermal Growth Factor (25 ng/ml), Hydrocortisone (0.5 µg/ml), Epinephrine (0.5 µg/ml), Transferrin (10 µg/ml), Insulin (5 µg/ml), Triiodothyronine (6.5 µg/ml), all-trans retinoic acid (50 nM), Bovine Pituitary Extract (52 µg/ml), Gentamicin (30 µg/ml), and Amphotericin B (15 ng/ml), all included in the SingleQuots Kit purchased by Clonetics (Lonza), with the exception of human recombinant epidermal growth factor and all-trans retinoic acid that were purchased by Sigma-Aldrich Co (St. Louis, MO, USA).

Primary cultures of human nasal epithelial cells were obtained from the explant method as previously outlined [34], [35], [36]. Briefly, tissue explants were carefully dissected away from the mucosal surface of tissue specimens by using sterile surgical material. Size of explants obtained was of approximately 20–30 mm^2^. Small explants were washed three times with fresh, sterile media, in order to remove blood and mucous when was present. Then, tissue explants were plated on 6-well plates coated with rat tail collagen type I (Sigma-Aldrich), always orientated with the epithelial layer to be in contact with the collagen-coated culture plates. A single drop of growth medium was added directly on to the tissue to allow the tissue to adhere to the plate. Once the tissue was adherent, more media was added slowly (in order not to dislodge the tissue). Explant culture was performed in a 5% CO_2_ humidified atmosphere at 37°C, with the culture media being changed daily. Cell growth from explants usually started from day 3 to day 7 (see Figure S1).

To demonstrate the purity of epithelial cells obtained from both NP and control NM explants, the cells were trypsinized using the Subculture Reagent Pack (Lonza). Cytospin slide preparations were fixed in acetone for 15 minutes, and processed for immunofluorescent detection of epithelial (keratin 18^+^) and mesenchymal (vimentin^+^) cell markers (see Figure S2).

### Submerged Cell Culture

When confluence was evident in the well, cells grown from NP and control NM explants were trypsinized using the Subculture Reagent Pack (Lonza). These cells were then cultured in 6-well plates coated with collagen type I using growth media. The culture was performed in a 5% CO_2_-humidified atmosphere at 37°C and the media was changed every 48 h during the submerged culture (see Figure S1).

### Mucociliary Differentiation of Human Nasal Mucosa and Polyp Epithelial Cells in Air-Liquid Interface (ALI)

After reaching 80–90% subconfluence in the submerged culture, epithelial cells were subcultured and seeded at a density of 5×10^4^ cells/cm^2^ onto 0.33 cm^2^ inserts with polyethylene terephthalate (PET) transparent membranes for growth of attachment-dependent cells without matrix (0.4 µm pore size, 24-well, Millipore, Billerica, MA, USA) and incubated at 37°C supplemented with 5% CO_2_. Cells were submerged in differentiation media composed of 50% DMEM (Lonza) in BEBM supplemented with Epidermal Growth Factor (0.5 ng/ml), Hydrocortisone (0.5 µg/ml), Epinephrine (0.5 µg/ml), Transferrin (10 µg/ml), Insulin (5 µg/ml), Triiodothyronine (6.5 µg/ml), all-trans retinoic acid (50 nM), Bovine Pituitary Extract (52 µg/ml), Gentamicin (30 µg/ml), and Amphotericin B (15 ng/ml) for the first 7 days.

Cells were then cultured for differentiation for a further 28 days with the apical surface exposed to air (cell culture in ALI). Throughout the culture period cells were maintained at 37°C in a 5% CO_2_ incubator with media changes every 48 hours (see Figure S1).

Mucociliary differentiation was evaluated at days 0, 7, 14, 21 and 28, corresponding to an initial step (day 0), intermediate steps of differentiation between step 2 (day 7, undifferentiated pluristratified epithelium) and step 5 (day 28, mature pseudostratified differentiated epithelium). The following methods were used: electron microscopy for the ultrastructural study, immunochemical techniques for histological and cell analyses, and enzyme-linked immunosorbent assay (ELISA) to assess mucous and serous secretion of cultured epithelia. Additionally, at day 28, cytokine and chemokine secretion from reconstituted epithelia from NP and control NM ALI cultures were measured by cytometric bead array (CBA, BD Biosciences, San Jose, CA, USA) in order to assess NP inflammatory function compared to control NM.

### Ultrastructutal Study by Electron Microscopy

Scanning Electron Microscopy (SEM) and Transmission Electron Microscopy (TEM) were performed on ALI cultures at days 0 (initial), 14 (intermediate) and 28 (final step).

For TEM, the cells on inserts were fixed with 3.5% glutaraldehyde, post-fixed in 1% osmium tetroxide, dehydrated in a progression of increasing ethanol concentrations up to 100% ethanol, and embedded in epoxy resin. Thick sections were obtained and stained with toluidine blue for light microscopy. Then, ultrathin sections from selected areas were produced by sectioning with ultramicrotome and collected in copper grids. Finally, sections were stained with lead citrate and uranyl acetate, and then examined with the transmission electron microscope, JEOL 1010 (JEOL, USA), equipped with Bioscańs camera (Gatan, USA) at Scientific-Technical Services at the Faculty of Medicine, Universitat de Barcelona.

For SEM, the cells on inserts were fixed with 3.5% glutaraldehyde, post-fixed in 1% osmium tetroxide, and dehydrated in a progression of increasing ethanol concentrations up to 100% ethanol. The samples were then critical-point dried in CO_2_, mounted on scanning electron microscope stubs, and sputter-coated with gold palladium alloy before examination under the microscope. The surface of ALI cultures were then examined with a scanning electron microscope, Zeiss DSM 940 A (Zeiss, Germany).

Preparation of samples and electron microscopy were carried out at Scientific-Technical Services at the Faculty of Medicine, Universitat de Barcelona.

### Histological Characterization by Immunocytochemistry

To characterize the mucociliary differentiation of NP and control NM ALI cultures, cells were fixed at different days (0, 7, 14, 21, and 28) with 4% paraformaldehyde in the membranes of inserts, and these were extracted from the plastic support and carefully cut in three small pieces for paraffin inclusion (the membrane pieces were oriented in the paraffin blocks to allow the obtention of cross sections of the ALI cultures). De-paraffinization in graded ethanol concentrations and microwave oven antigen retrieval (350 W, 4–5 min, in pH 6.6 citrate buffer) were performed on 5 µm sections of paraffin-embedded cell culture. Subsequently, samples were differentially processed depending on the detection method used.

For colorimetric detection, the indirect immuno-peroxidase technique was performed. Samples were blocked with 3% hydrogen peroxide in order to reduce background staining prior to incubation overnight at 4°C with primary antibodies, including mouse monoclonal anti-ΔNp63 (4A4, Santa Cruz Biotechnology, Dallas, TX, USA) for basal progenitor/stem cells, mouse monoclonal anti-MUC5AC (45M1, Thermo Fisher Scientific, Waltham, MA, USA) for goblet cells, and mouse monoclonal anti-β-Tubulin IV (clone ONS.1A6, Sigma-Aldrich, St. Louis, MO, USA) for ciliated cells. Negative controls were performed by omitting the primary antibody and using a negative control serum of the same isotype. The EnVision + System-HRP (DAB, Dako, Glostrup, Denmark), was used for mouse primary antibody labelling. This was conducted in accordance with the manufacturer’s instructions for the purposes of colorimetric detection. Finally, immunocytochemically-stained sections of NM and NP ALI cultures were examined with the Olympus BX41 light microscope (Olympus Corporation, Tokio, Japan), and total and positive cells were counted in three wells (5 fields from different areas of each well at 400x magnification) from different nasal polyp (NP, n = 3) and control nasal mucosa (NM, n = 3) ALI cultures. The results were subsequently averaged.

For immunofluorescent detection, the cells were permeabilized with 0.2% Triton X-100 (Sigma-Aldrich Co.) in phosphate buffered saline, pH 7.4 (PBS, Sigma-Aldrich Co.), at room temperature, and then blocked with 6% goat serum (Sigma-Aldrich Co.) for 60 minutes to prevent unspecific binding. Incubation overnight at 4°C with primary antibodies, including rabbit polyclonal antibody anti-MUC5B (LUM5B.2) [37] for goblet cells, and mouse monoclonal anti-β-Tubulin IV (clone ONS.1A6, Sigma- Aldrich) for ciliated cells, mouse monoclonal anti-Cytokeratin 18 (Clone DC 10, DAKO) for epithelial cells, and mouse monoclonal anti-Vimentin (Clone Vim 3B4, DAKO) for mesenchymal cells. Negative controls were performed by omitting the primary antibody and using a negative control serum of the same isotype. Alexa Fluor 488 Goat Anti-Mouse IgG (H+L, green channel), Alexa Fluor 594 Goat Anti-Mouse IgG (H+L, red channel), and Alexa Fluor 594 Donkey Anti-rabbit IgG (H+L, red channel) were used as the secondary antibodies (Invitrogen, Life Technologies Ltd, UK). The samples were counterstained with DAPI to identify cell nuclei (blue channel), and the slides were cover-slipped with ProLong Gold Antifade Reagent (Invitrogen, Life Technologies Ltd, UK). Finally, slides were examined with the Leica DMI6000 B inverted microscope (Leica Microsystems, Germany).

### Measurement of Mucous and Serous Secretion in Apical Washes

At different times (days 0, 7, 14, 21, and 28), apical surfaces from NP and control NM ALI cultures were rapidly washed twice with warmed PBS (500 µL) to mimic nasal lavage, and stored at −80°C for further analysis. MUC5AC, MUC5B, and lactoferrin levels were detected in washing samples by using ELISA. Apical washings were incubated at 37°C for 90 minutes on MaxiSorp microtiter 96-well plates (Nunc, Rochester, NY, USA). After washing with PBS-0.05% Tween-20 (PBS-T, Sigma-Aldrich), wells were blocked for 1 hour at 37°C with 1% bovine serum albumin (BSA; Sigma-Aldrich) in PBS-T for MUC5AC, 1% gelatine (Sigma-Aldrich) in PBS-T for MUC5B, or 6% goat serum in PBS-T for lactoferrin. After three washes with PBS-T, wells were sequentially incubated for 1 hour at 37°C with the primary antibodies: mouse monoclonal anti-MUC5AC (45M1, Thermo Fisher Scientific), rabbit polyclonal anti-MUC5B (LUM5.B2) [37], and rabbit polyclonal anti-lactoferrin (Abcam, Cambridge, UK). After washing with PBS-T, samples were incubated for 1 hour at 37°C with HRP-conjugated horse anti-mouse, or HRP-conjugated goat anti-rabbit antibody (Vector Laboratories, Burlingame, CA, USA). After washing, 3,3′, 5,5′-tetramethylbenzidine substrate (Pierce, Rockford, IL, USA) was added and incubated in the dark for 15 minutes at room temperature, followed by stop solution (2N H_2_SO_4_) to terminate the reaction. Absorbance was read at 450 nm on a Multiskan EX (Thermo Electron Corporation, Vantaa, Finland). The relative amount of MUC5AC, MUC5B, and lactoferrin in each sample was determined from their A_450_ values using standard curves constructed with serial dilutions of the commercial mucins from porcine stomach type II (Sigma-Aldrich), human saliva [38], and lactoferrin from human milk (Sigma-Aldrich) respectively. Results were expressed as percentages compared to day 0 (100%) of ALI culture. Sample and standard duplicates showed a variation rate of <5%.

### Quantitative Analysis of Cytokine and Chemokine Production

At days 0 (initial), 14 (intermediate) and 28 (final step), apical surfaces from NP and control NM ALI cultures were rapidly washed twice with warmed PBS (500 µL) to mimic nasal lavage, and stored at −80°C for cytometric bead array analysis (CBA) of different cytokines and chemokines. The Human Inflammatory Cytokine and Human Chemokine Kits, and GM-CSF and Eotaxin 1 Flex Sets (BD Biosciences) were used according to the manufacturer’s instructions to quantitatively measure the following proteins: interleukin-8 (IL-8), interleukin-1β (IL-1β), interleukin-6 (IL-6), interleukin-10 (IL-10), tumor necrosis factor alfa (TNFα), and interleukin-12p70 (IL-12p70), RANTES (CCL5/RANTES), monokine induced by interferon-γ (CXCL9/MIG), monocyte chemoattractant protein-1 (CCL2/MCP-1), and interferon-γ–induced protein-10 (CXCL10/IP-10), eotaxin 1, and granulocyte-macrophage colony-stimulating factor (GM-CSF). Briefly, 50 µl of unknown samples or recombinant standards were added to 50 µl mixed capture beads and incubated for 3 hours with 50 µl of phycoerythrin-conjugated detection antibodies (Ab-PE) to form sandwich complexes. After washing to remove unbound Ab-PE detection reagent, and acquiring samples on a flow cytometer FACSArray (BD), results were generated using FCAP Array software (BD).

### Vídeo Record of Ciliated Cells

AVI video was recorded with an Olympus Fe 5020 camera connected to light microscopy at 400x magnification (See Video S1).

### Statistical Analysis

Data are reported as median and 25–75th interquartiles, for the different day analyses, for each cell marker immunocytochemical outcome (percentages of positive cells from total number of cells), for MUC5AC (ng/ml), for MUC5B (RU/ml, relative units/ml was determined relative to standard saliva from one healthy person), for lactoferrin secretion (ng/ml), and for cytokine and chemokine production (pg/ml).

The statistical analysis was performed using SigmaPlot Version 10.0 software (Systat, Chicago, IL) and PASW Statistics 18.0 (IBM Corp., Armonk, NY). The nonparametric Wilcoxon Signed-Ranks Test was used for paired comparisons (when comparing days 7, 14, 21, 28 vs day 0), and Mann-Whitney U test was used for between-group comparisons (when comparing NP vs control NM at different days). Statistical significance was set at p<0.05.

## Results

### Ultrastructural Study

Mucociliary differentiation process of epithelial cells from both NP (n = 3) and control NM (n = 3) in the ALI culture was analyzed by scanning and transmission electron microscopy at days 0, 14, and 28.

**Figure 1:**
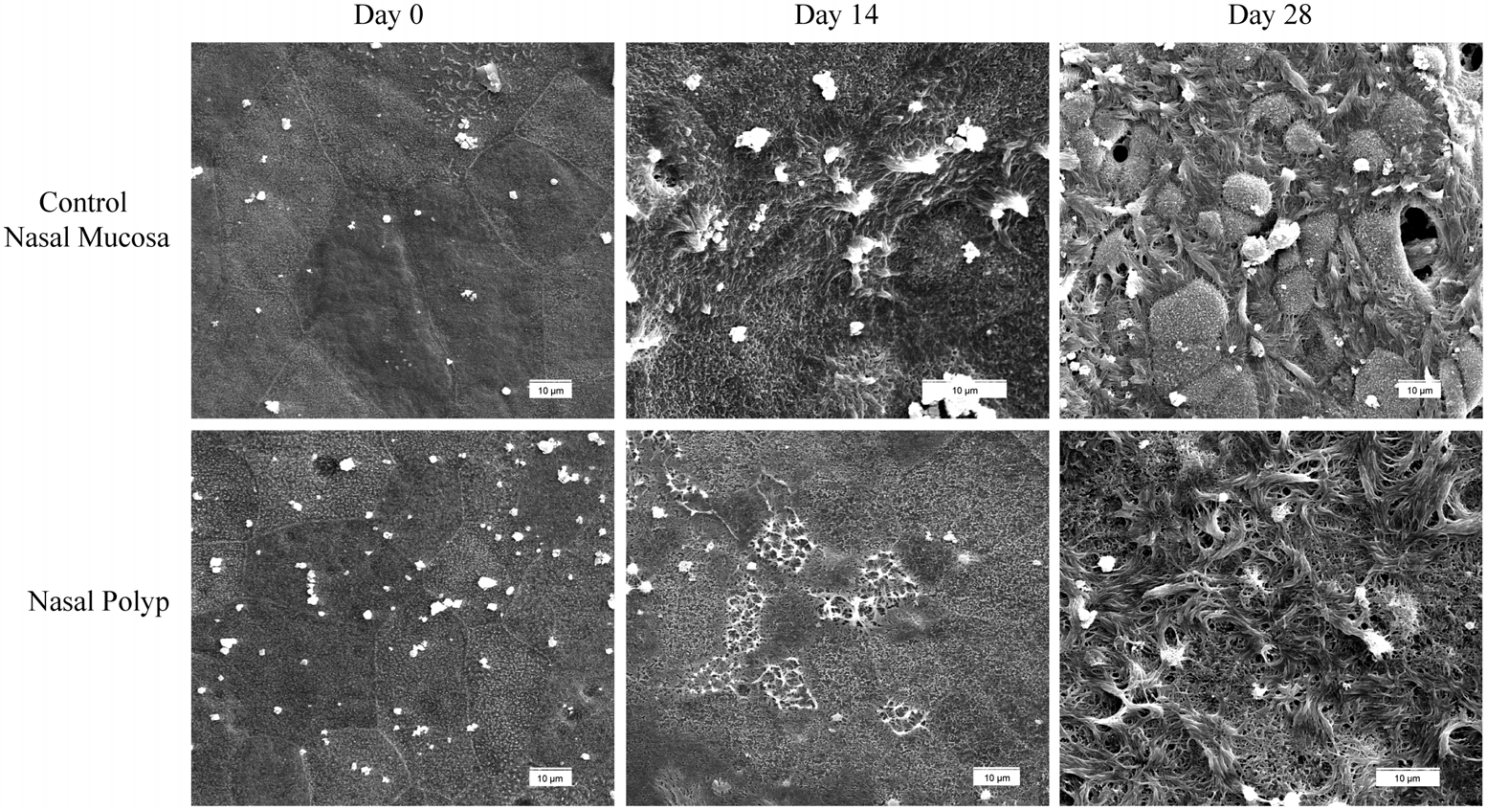
Ultrastructural study of apical surfaces in ALI cultures from both nasal polyp and control nasal mucosa at different days by scanning electron microscopy. At day 0 (left column), representative images are showing apical surfaces without ciliated cells. At days 14 (centre column) and 28 (right column), apical surfaces showing ciliated cells.

Apical surfaces from both NP and control NM ALI cultures were observed by scanning electron microscopy (Figure 1). At day 0 apical surfaces showed tightly packed epithelial cells, without presence of ciliated cells. Whereas at days 14 and 28, apical surfaces from cultures clearly showed the presence of ciliated cells in both NP and control NM, as well as the presence of ciliated cells, with the length of cilia being higher at day 28 than at day 14 (Figure S3, A and B). On the other hand, non-ciliated cells were showing microvilli on their apical surface at the different analysed times (Figure S3, A and B).

**Figure 2:**
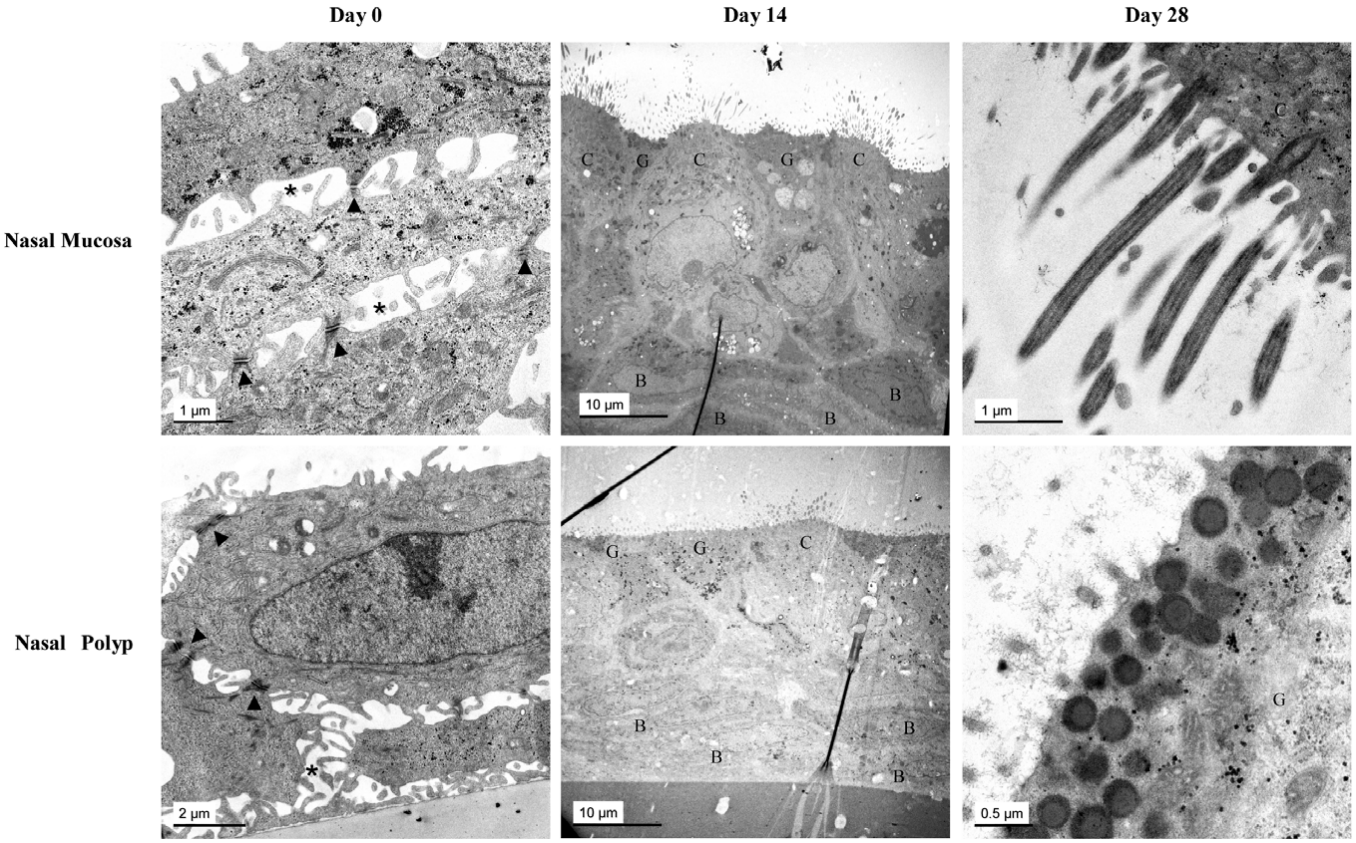
Ultrastructural study in ALI cultures from both nasal polyp and control nasal mucosa at different days by transmission electron microscopy. At day 0 (left column), representative images are showing desmosomes (arrowheads), interdigitations (asteriks). At day 14 (centre column), ciliated (C), goblet (G) and basal (B) cells. At day 28 (right column), longitudinal sections of motile cilia (upper) and secretory granules of a goblet cell (lower).

Ultrastructure of epithelial cells from both NP and control NM ALI cultures were also examined by transmission electron microscopy (Figure 2). At day 0, non-polarized epithelial cells were observed in both NP and control NM cultures, displaying intercellular spaces between cells, but being connected by desmosomes and inter-digitations. In addition, neither ciliated nor goblet cells were observed in both NP and control NM ALI cultures. In contrast, at days 14 and 28, epithelial cells formed a pseudostratified, polarized, and fully differentiated epithelium in both NP and control NM ALI cultures, which included ciliated, goblet, and basal cells. At days 14 and 28 cells were joined by apical junctional complexes, formed by tight junctions, adherens junctions, and desmosomes, showing a tightly packed epithelium without intercellular spaces (Figure S3, C and D). In both NP and control NM reconstituted epithelia, ciliated cells were interspersed with goblet cells and non-ciliated columnar cells, and orientated to apical side, while basal cells were located at the basal side. Motile cilia were observed on the apical surface of ciliated cells, attached to cells by basal bodies, and showing in cross-sections the typical axonemes, with the latter being composed of central microtubule singlets in addition to the nine outer doublets (9+2) (Figure S3, E and F). Goblet cells revealed secretory vesicles, with these being located along with non-ciliated columnar cells, covered by microvilli and surrounded by the glycocalix (Figure S3, G and H).

At the ultrastructural level, no differences were found when comparing NP with control NM ALI cultures.

### Histological Characterization

**Figure 3:**
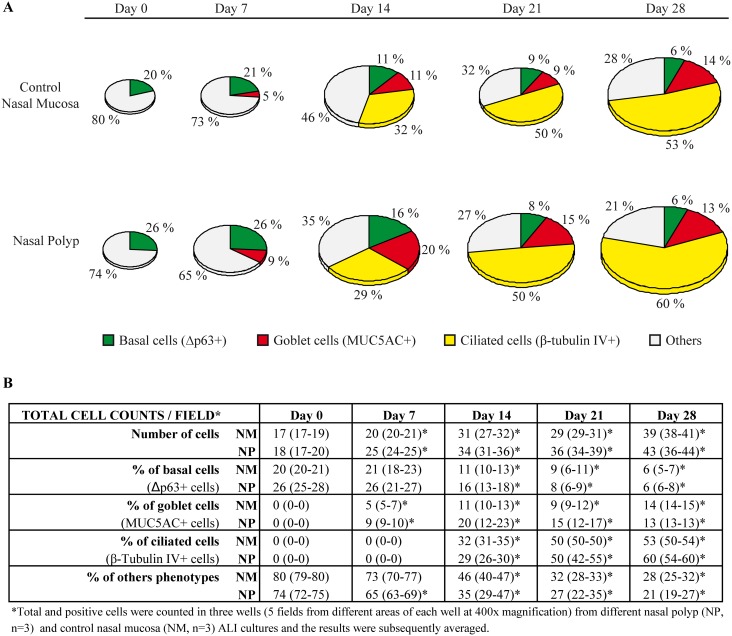
Re-differentation over time of human nasal epithelial cells from both nasal polyp and control nasal mucosa in ALI cultures. A, Pie charts, with modeled size relative to total cell number, are representing the percentage of different cell phenotypes over time. B, table shows the total number of cells and percentages of cell phenotypes from both cultures types. Total and positive cells were counted in three wells (5 fields from different areas of each well at 400x magnification) from different nasal polyp (NP, n = 3) and control nasal mucosa (NM, n = 3) ALI cultures and the results were subsequently averaged. *, P<0.05 vs day 0. The nonparametric Wilcoxon Signed-Ranks Test and Mann-Whitney U test were used for paired comparisons and between-group comparisons respectively. Statistical significance was set at P<0.05.

The re-differentiation process from day 0 to day 28 was characterized by immunocytochemical detection of different cell phenotypes, at different culture times (0, 7, 14, 21, and 28 days), in both NP (n = 3) and control NM (n = 3) ALI cultures. Histological analysis of both culture types showed that, primary epithelial cells grew from monolayers, including basal progenitor/stem cells (ΔNp63^+^), to form well-differentiated epithelia, including goblet cells (MUC5AC^+^), ciliated cells (β-tubulin IV^+^), and basal progenitor/stem cells (ΔNp63^+^) (Figure 3, A and B).

**Figure 4:**
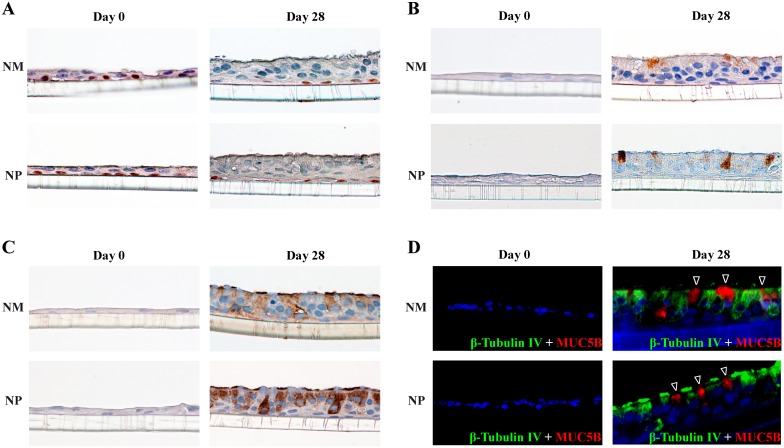
Representative immunocytochemical images of different cell phenotypes in both undifferenciated (day 0) and fully differentiated (day 28) cultures from both nasal polyp and control nasal mucosa. A, Basal progenitor/stem cells, with positive immunoreactivity to ΔNp63. B, Goblet cells, with positive immunoreactivity to MUC5AC. C, Ciliated cells, with positive immunoreactivity to β-tubulin IV. D, Ciliated cells, marked by β-tubulin IV^+^ (green), and goblet cells, marked by MUC5B^+^ (red, arrowheads). Final magnification x400.

The number of cells increased significantly (P<0.05) at days 7, 14, 21, and 28, in both NP and control NM cultures compared to day 0, but without significant differences between both cultures (Figure S4). Basal progenitor/stem cells (ΔNp63+, Figure 3, 4A) were always observed in both NP and control NM cultures, whereas Goblet cells (MUC5AC^+^, Figure 3, 4B), and ciliated cells (β-tubulin IV^+^, Figure 3, 4C, 4D) were only observed from day 7 and day 14, respectively. Compared to day 0, basal progenitor/stem cells (ΔNp63+) were decreased significantly (P<0.05) at days 14, 21, and 28, while goblet cells (MUC5AC^+^), and ciliated cells (β-tubulin IV^+^) were increased significantly (P<0.05) from day 7 and day 14, respectively (Figure 3B).

Co-immunofluorescence demonstrated that, in both fully differentiated cultures from both NP and control NM, ciliated (β-tubulin IV^+^) and goblet (MUC5B^+^) cells were clearly present as distinct cell phenotypes (Figure 4D).

At the cell phenotype level, no significant differences were found when comparing NP with control NM at 0, 7, 14, 21, or 28 days of ALI cultures.

### Mucous and Serous Secretion

**Figure 5:**
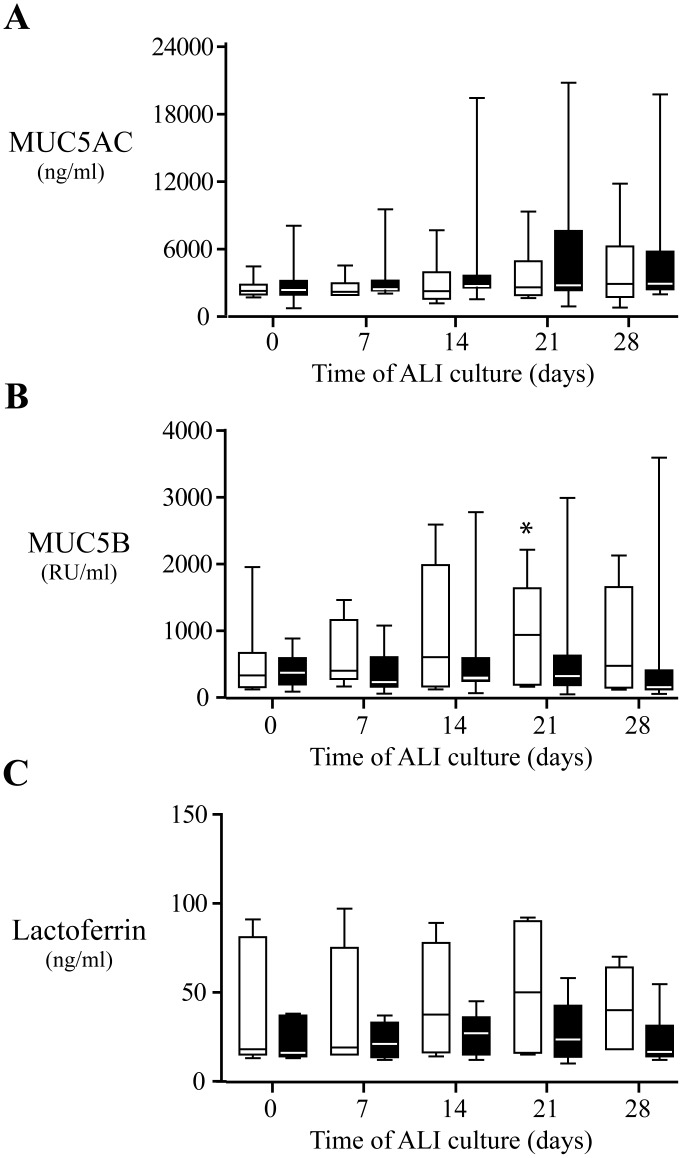
Overtime secretion of mucin and lactoferrin in nasal polyp (black boxes) and control nasal mucosa (white boxes) ALI cultures. A, MUC5AC secretion (ng/ml). B, MUC5B secretion (RU/ml). C, Lactoferrin secretion (ng/ml). Results are expressed as medians and interquartile ranges with 25^th^–75^th^ percentiles. *, P<0.05 vs day 0. Statistical analysis by Mann-Whitney U test (between groups) and by Wilcoxon test (within groups).

The secretion of MUC5AC, MUC5B, and lactoferrin was assessed by ELISA in apical washes of both NP (n = 9) and control NM (n = 7) ALI cultures from day 0 to day 28 (Figure 5).

Compared to day 0, no significant differences were found in MUC5AC, MUC5B, and lactoferrin, secretions overtime in both NP and control NM ALI cultures, with the exception of MUC5B that was significantly (P<0.05) increased in control NM ALI culture at day 21 (927, 178–1624 RU/ml) compared to day 0 (318, 143–655 RU/ml) (Figure 5B).

At days 7, 14, 21, and 28 of ALI culture, secretions overtime of MUC5B and lactoferrin, but not MUC5AC, tended to be lower in NP ALI cultures than in control NM ALI cultures, but without reaching statistical significance (Figure 5).

### Cytokine and Chemokine Production

**Figure 6:**
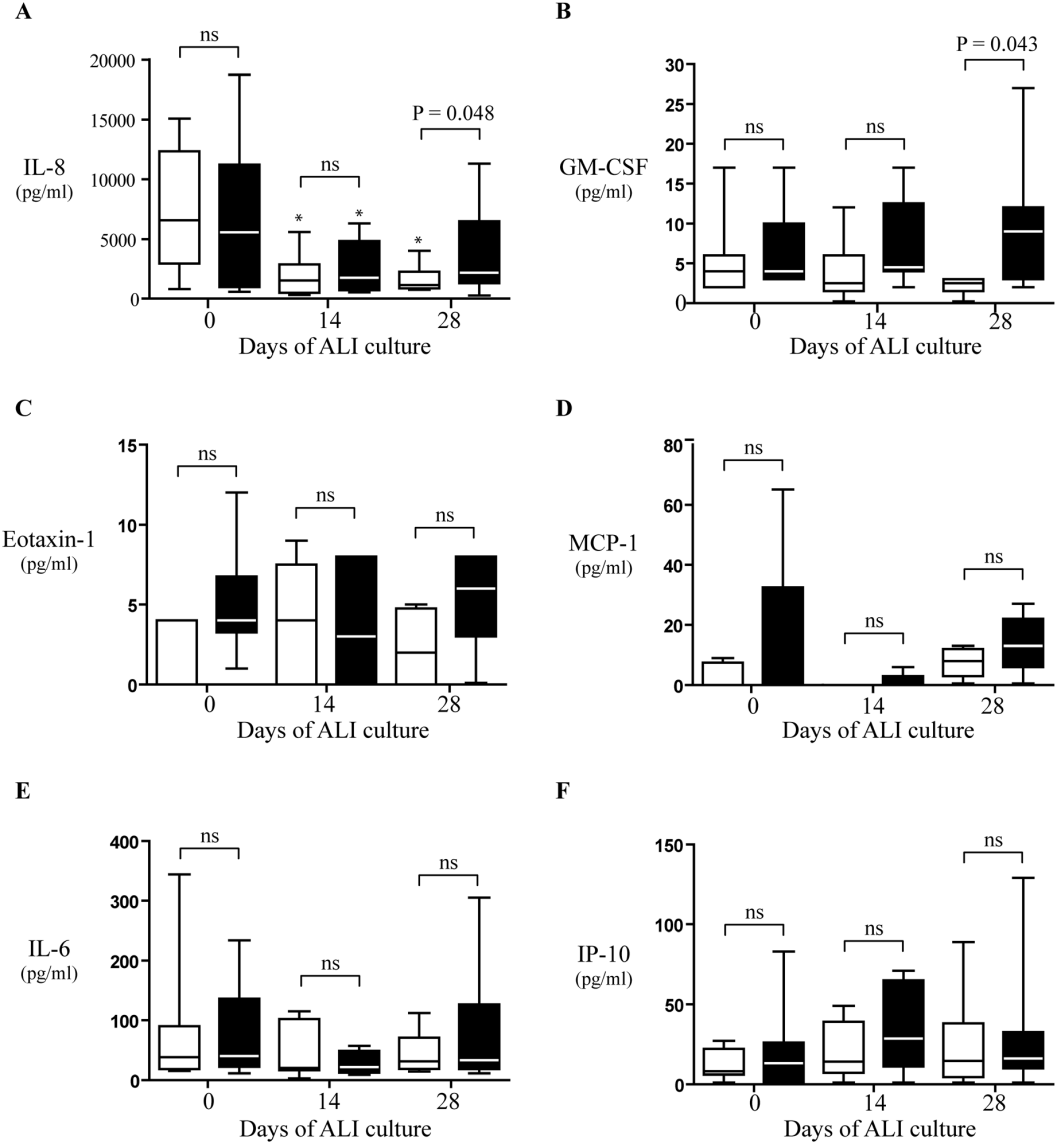
Cytokine and chemokine apical secretion from both nasal polyp (black boxes) and control nasal mucosa (white boxes) ALI cultures at different days. A, interleukin-8 (IL-8), B, granulocyte-macrophage colony-stimulating factor (GM-CSF), C, eotaxin 1, D, monocyte chemoattractant protein-1 (MCP-1), E, interleukin-6 (IL-6), and F, interferon-γ–induced protein-10 (IP-10), levels in apical washes. Results are expressed as medians and interquartile ranges with 25th and 75th percentiles. ns, P>0.05 vs day 0 (100%); *, P<0.05 vs day 0. Statistical analysis by Mann-Whitney U test (between groups) and by Wilcoxon test (within groups).

At days 0, 14, and 28, a number of cytokine (IL-8, IL-1β, IL-6, IL-10, TNF-α, and IL-12p70) and chemokine (RANTES, MIG, MCP-1, IP-10, eotaxin-1, and GM-CSF) were analyzed by using CBA to compare levels in wash samples of the apical surfaces of NP (n = 9) and control NM (n = 7) ALI cultures. IL-8, IL-6, eotaxin-1, GM-CSF, IP-10, and MCP-1 were detected in both epithelial secretions (Figure 6), whereas IL-1β, IL-10, TNF-α, IL-12p70, RANTES, and MIG were found to be below detection limit.

Among the detected cytokines and chemokines, during mucociliar re-differentiation process, at days 0 (initial step) and 14 (intermediate step), no significant differences were found when comparing NP with control NM ALI cultures (Figure 6). However, in well-differentiated epithelium, at day 28, the following differences were found: IL-8 and GM-CSF levels were significantly (P<0.05) greater in NP apical secretions than in control NM (Figure 6, A and B); eotaxin-1 and MCP-1 levels had also increased in apical secretions from NP, but without reaching statistical significance NM (Figure 6, C and D); and IL-6 and IP-10 levels were similar in NP and control NM epithelia (Figure 6, E and F).

## Discussion

In the present study we have developed and characterized the mucociliary differentiation over time of human NP epithelial cells in ALI culture system, compared to control NM. We have demonstrated: 1^st^) the mucociliary differentiation of NP epithelial cells in ALI system was similar to control NM at different analyzed days (0, 7, 14, 21, and 28); 2^nd^) NP reconstituted epithelia were showing similar histological features than control NM reconstituted epithelia; 3^rd^) secretion of IL-8 and GM-CSF was significantly increased in NP compared to control NM reconstituted epithelia, these findings being in keeping with previous studies [1], [20], [39]. Overall, our findings provide evidence that 3D *in vitro* model of NP reconstituted epithelium maintain an inflammatory function, assessed by secretion of cytokines and chemokines, which is characteristic of CRSwNP tissue.

At the initial step of mucociliary differentiation, i.e. day 0 of ALI culture, we detected in both NP and control NM cultures a population of basal epithelial stem cells (ΔNp63^+^), which decreased over time. The antibody used (clone 4A1, Santa Cruz) detects the ΔN isoform of p63 (ΔNp63), which has been identified as basal stem/progenitor cell marker in human [27], [40]–[42] and rodent [40], [41], [43], [44] tracheobronchial epithelium, as well as in human nasal epithelium [21], [45]. It has been reported that basal stem/progenitor cells are implicated in the regeneration of human tracheobronchial [42], [46] and nasal [21], [45] epithelium. Therefore, the reconstitution of both NP and control NM epithelia in our ALI cultures may be attributed, at least in part, to the identified ΔNp63^+^ basal stem/progenitor population. In fact, in both NP and control NM ALI cultures, ΔNp63^+^ cells and undifferentiated epithelial cells decreased during mucociliary differentiation, whereas goblet and ciliated cells were seen to be increasing from day 7 and day 14, respectively. These findings are in accordance with previous studies describing the mucociliary differentiation of normal human tracheobronchial [26], [29], [47] and partly with nasal [22], [23], [48] epithelial cells. In our study, neither ciliated nor goblet cells were observed at day 0 after confluence in both NP and control NM ALI cultures. In contrast, studies from Yoon et al. [22] and Kim et al. [23] demonstrated the presence of ciliated and goblet cells at day 2 after confluence in ALI cultures performed with normal human nasal epithelial (NHNE) cells. These differences could be produced by the different origin of cells used, different techniques and antibodies used for detection of both cell phenotypes, and the different days analyzed. On the other hand, comparing NP with control NM ALI cultures, we did not detect differences in the cell phenotype percentages during mucociliary differentiation.

In airway mucosa the epithelium acts as physical barrier and contributes to the innate immune system by secretion of mucus and antimicrobial peptides [7]. In the present study we have provided evidence that, in both NP and control NM ALI cultures at day 0, epithelial cells were connected by desmosomes and inter-digitations, but not linked by complete apical junctional complexes, composed of tight junctions, adherens junctions, and desmosomes [49], [50]. These cell junctions are multiprotein complexes that play a role in maintaining the integrity, polarity and functionality of the airway epithelial barrier [49]–[52]. Thus, absence of apical junctional complexes at day 0 suggests that epithelial cells probably were not apically-basally polarized. In contrast, at days 14 and 28, epithelial cells were joined by complete apical junctional complexes and clearly showing an apically-basally polarized epithelia in both NP and control NM ALI cultures. On the other hand, we elected to focus on mucins MUC5AC and MUC5B because they are the two major components in the mucus of human airways [53], [54], and lactoferrin, due to it being one of the most abundant airway antimicrobial peptides [7]. Despite not reaching statistical significance, we found that mucous (MUC5AC and MUC5B) and serous (lactoferrin) secretions were slightly increased in both cultures. The increased mucus production during mucociliary differentiation is probably due to the increased number of goblet cells, which were expressing both mucins in both cultures. These findings are partly in keeping with previous studies that have reported an increased mucin expression during differentiation of nasal epithelial cells in ALI culture [19], [22], [48]. On the other hand, we also observed that MUC5B and lactoferrin secretions tended to be lower in NP compared to control NM ALI cultures. Since it has been described that submucosal glands [55], [56] and also certain antimicrobial proteins, most notably lactoferrin [57], [58], are diminished in CRSwNP, our results for the MUC5B and lactoferrin secretions could be indicating that there is a glandular phenotype in control NM ALI cultures, that may be not well developed during the mucociliar differentiation of NP epithelial cells. Nevertheless, although we detected decreased levels of MUC5B and lactoferrin in NP compared to control NM, we are speculating because differences between both cultures did not reach statistical significance. Obviously, further studies are necessary to explore and confirm that epithelial cells have a glandular phenotype, or even if they are able to form glands, when cultured at the ALI system.

In contrast to the similarities in mucociliary differentiation of both NP and control NM ALI cultures, we found relevant differences associated with inflammatory function of CRSwNP epithelium, when cytokine (IL-8) and chemokine (GM-CSF) production were analyzed in well-differentiated epithelium. Our findings are in keeping with previous studies that have reported the increase of pro-inflammatory cytokines, such as IL-8 [39], [59], [60] and chemokines, as GM-CSF [39], [61], [62], eotaxin [63], and MCP-1 [64] in CRSwNP, compared to control nasal mucosa. These cytokines and chemokines have been implicated in eosinophil inflammation in nasal polyps [1], [39], [62], [63] and are downregulated with glucocorticoid [39], [62], [65], [66] or anti-leukotriene [67] treatments. Furthermore, our results from apical washes, particularly those for IL-8 and GM-CSF, are in keeping with the literature describing increased levels of pro-inflammatory mediators in nasal lavages from CRSwNP patients compared to controls [68], [69]. The present study therefore provides direct evidence that fully differentiated epithelium obtained from NP epithelial cells may sustain an inflammatory function associated to CRSwNP by secreting higher levels of cytokines and chemokines than control NM epithelia.

However, there are limitations to our study. First, some of our results may be have a low reproducibility due to we detected great variability, for instance, in the mucus and lactoferrin secretions. Moreover, the mucociliar differentiation of epithelial cells may varies between cultures from different donors, different wells, or even in different areas of the well. For instance, the presence of ciliated cells varies between different areas of the well. Secondly, we have not seen in the NP reconstituted epithelium the typical epithelial alterations of the sinus mucosa from patients with CRSwNP, where altered epithelium shows goblet or basal cell hyperplasia, or metaplasia [2]. This could be due, at least in part, to the absence of inflammatory cells and the release of their inflammatory mediators in our ALI culture. Exposure of NP ALI cultures to inflammatory stimuli could potentially induce similar epithelial alterations as in the sinus mucosa of patients with CRSwNP. In fact, previous reports have demonstrated that in ALI cultures from normal human bronchial [70]–[72] and guinea pig tracheal [31], [73] epithelial cells, the prolonged exposition to IL-13 induces goblet cell hyperplasia in culture. Nevertheless, to our knowledge, this is the first study demonstrating an elevated cytokine and chemokine secretion at basal level from NP reconstituted epithelia in ALI system. It could be relevant because provide more evidence that, as previously suggested by other studies [20], [21], the 3D *in vitro* model of NP regenerated epithelium may be is a good model of the CRSwNP epithelium.

Finally, future studies should investigate the proliferation, maintainenance, and differentiation of ΔNp63^+^ basal cells, which were identified in our study, and have been recently described as adult tissue stem cells of nasal epithelium [45]. The biology and relevance of stem cells in nasal epithelium remain unclear. Identification of molecular mechanisms of nasal stem cells involved in repair, proliferation, and mucociliary differentiation under normal and pathological conditions, provides the potential for the development of new strategies for CRSwNP treatment.

## Conclusion

Reconstituted epithelia from human nasal polyp epithelial cells cultured in ALI system provides a *3D in vitro* model, more homogeneous, standarized, and reproducible than organotypic explant cultures, that could be useful for repair and differentiation, as well as physiopathological, pharmacological, toxicological, and transport studies (Table 1). Moreover, NP reconstituted epithelium provides a model that could be useful for studying the role of epithelium in CRSwNP and for developing new therapeutic strategies for CRSwNP, including cell therapy.

## Supporting Information

Figure S1 **Scheme of the different epithelial cell primary culture steps.** Epithelial cells from both nasal mucosa and nasal polyps were obtained after 5–7 days of explant culture in appropriate culture medium. During 1 week epithelial cells proliferate in a regular submerged culture, being then sub-cultured in inserts where cells were maintained in air-liquid interface culture for 28 days to obtain a well-differentiated, polarized, and pseudostratified airway epithelium.
(TIF)

Figure S2 **Characterization of human airway epithelial cells obtained from both control nasal mucosa and nasal polyp explants.** Confirmation of epithelial cell identity and purity by immunocytochemistry of cytospin preparations using cell type-specific markers: cytokeratin 18 (green) for epithelial cells and vimentin (green) for mesenchymal cells. DAPI (blue) was used to stain cellular nuclei. Epithelial cells isolated from both nasal mucosa and nasal polyps showed positive immunoreactivity to cytokeratin 18 (center column) and negative immunoreactivity to vimentin (right column). Contol cells showed no labeling in the absence of the primary antibody (left column).
(TIF)

Figure S3 **Representative images of ultrastructural study of nasal polyp ALI culture at days 14 and 28 by electron microscopy.** A–B, Representative images showing cilia in the apical surfaces by scanning electron microscopy. C–D, Apical junctional complexes, composed of tight junctions, adherens junctions, and desmosomes, linking two neighboring cells by transmission electron microscopy (TEM). E–F, Basal bodies of motile cilia (showing their typical structure of nine microtubule triplets) and cross-sections of cilia (showing typical axonemes, rings of nine outer microtubule doublets with two central microtubule doublets, 9+2) by TEM. G–H, Microvilia, glycocalix surrounding microvilli projections, and secretory granules by TEM.
(TIF)

Figure S4 **Epithelial cell counts of nasal polyp and control nasal mucosa ALI cultures at the different days analyzed.** Cell counts from different nasal polyp (filled circles, n = 3) and control nasal mucosa (n = 3) ALI cultures. Results are expressed by dots (media for three replicates of each culture) and line (median of three different cultures). ns, non-significant, statistical analysis by Mann-Whitney U test (between groups).
(TIF)

Video S1 Video is showing the synchronized cilia movement of ciliated cells from a mature and well-differentiated NP epithelium. This video shows that cilia are not only present in culture but also functional. The AVI video was recorded with an Olympus Fe 5020 camera connected to light microscopy and focusing at 400x magnification.
(AVI)
