# Supplementary figures and images for: Reconstituted Human Upper Airway Epithelium as 3-D In Vitro Model for Nasal Polyposis
Source: PLoS One. 2014 Jun 19;9(6):e100537. doi: 10.1371/journal.pone.0100537 (PMC4063947; doi:10.1371/journal.pone.0100537)

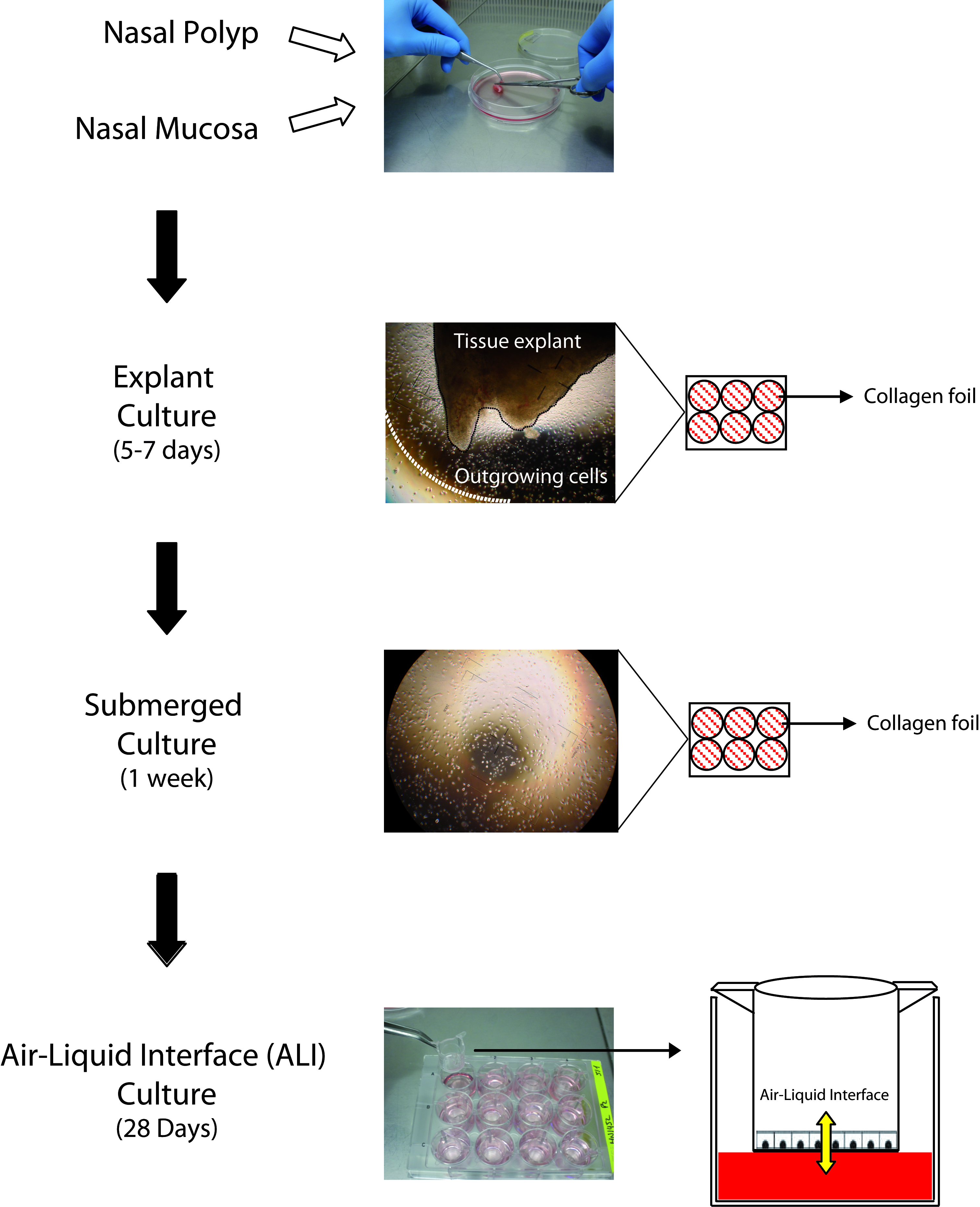

Supplement: Figure S1 — Scheme of the different epithelial cell primary culture steps. Epithelial cells from both nasal mucosa and nasal polyps were obtained after 5–7 days of explant culture in appropriate culture medium. During 1 week epithelial cells proliferate in a regular submerged culture, being then sub-cultured in inserts where cells were maintained in air-liquid interface culture for 28 days to obtain a well-differentiated, polarized, and pseudostratified airway epithelium. (TIF) [file pone.0100537.s001.tif]

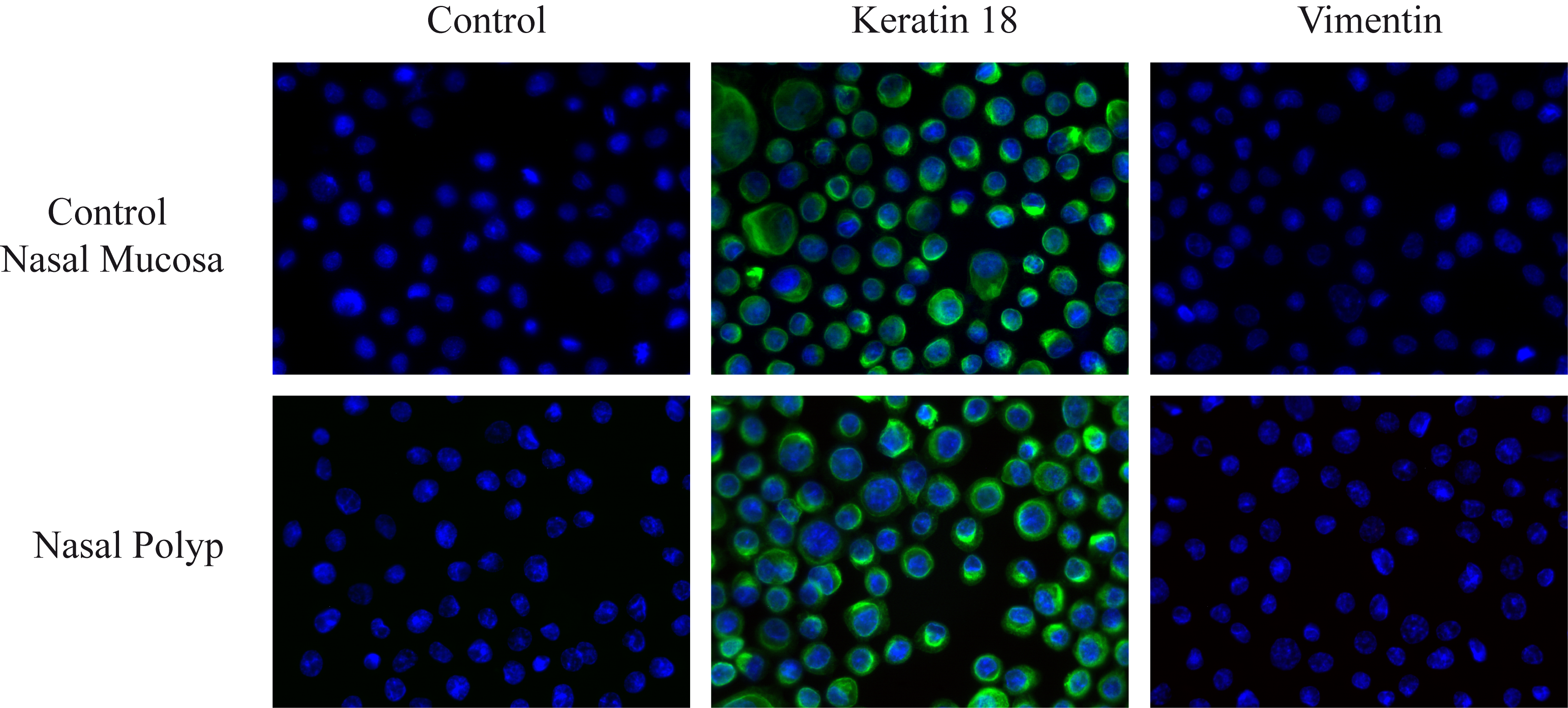

Supplement: Figure S2 — Characterization of human airway epithelial cells obtained from both control nasal mucosa and nasal polyp explants. Confirmation of epithelial cell identity and purity by immunocytochemistry of cytospin preparations using cell type-specific markers: cytokeratin 18 (green) for epithelial cells and vimentin (green) for mesenchymal cells. DAPI (blue) was used to stain cellular nuclei. Epithelial cells isolated from both nasal mucosa and nasal polyps showed positive immunoreactivity to cytokeratin 18 (center column) and negative immunoreactivity to vimentin (right column). Contol cells showed no labeling in the absence of the primary antibody (left column). (TIF) [file pone.0100537.s002.tif]

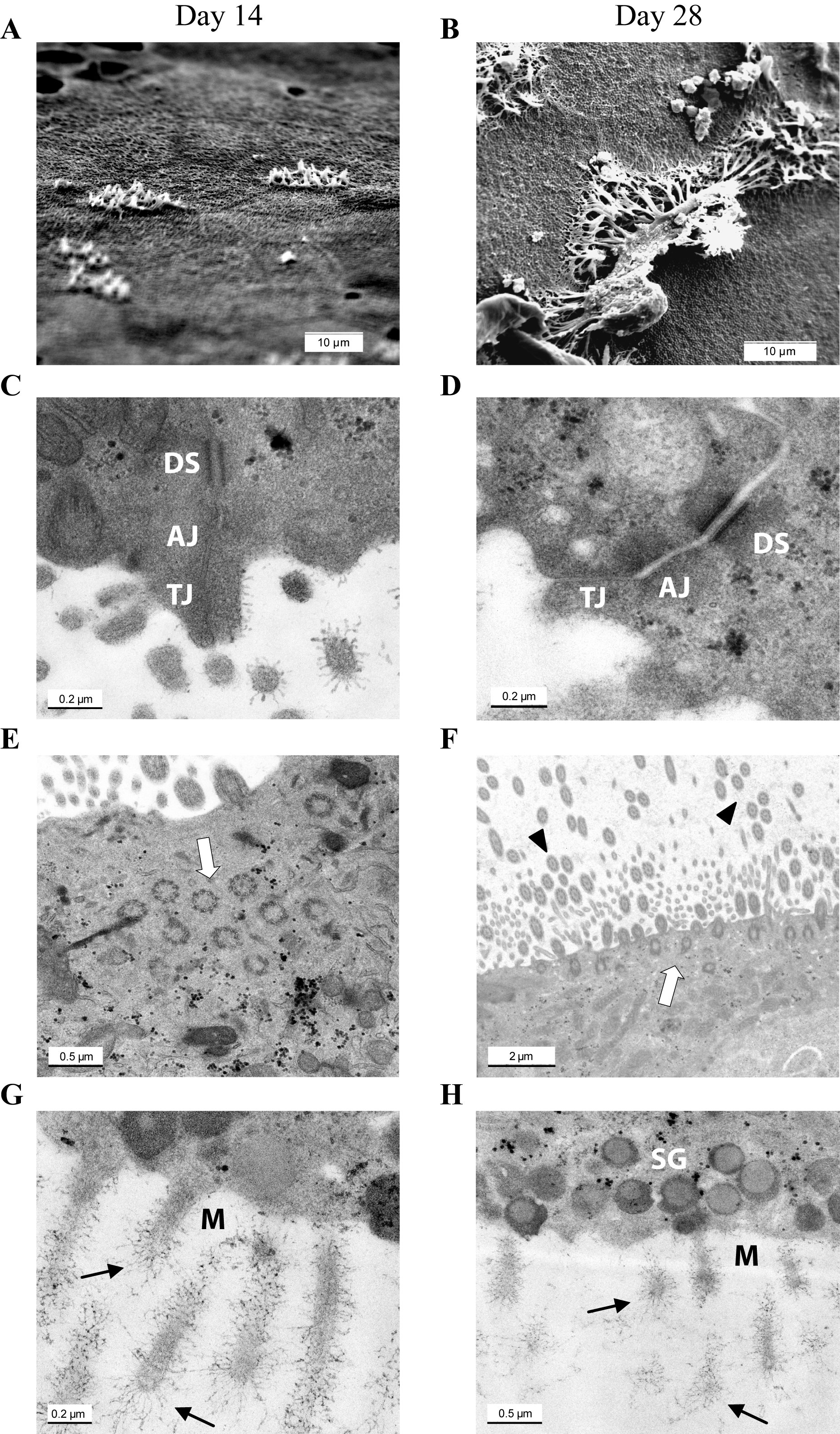

Supplement: Figure S3 — Representative images of ultrastructural study of nasal polyp ALI culture at days 14 and 28 by electron microscopy. A–B, Representative images showing cilia in the apical surfaces by scanning electron microscopy. C–D, Apical junctional complexes, composed of tight junctions, adherens junctions, and desmosomes, linking two neighboring cells by transmission electron microscopy (TEM). E–F, Basal bodies of motile cilia (showing their typical structure of nine microtubule triplets) and cross-sections of cilia (showing typical axonemes, rings of nine outer microtubule doublets with two central microtubule doublets, 9+2) by TEM. G–H, Microvilia, glycocalix surrounding microvilli projections, and secretory granules by TEM. (TIF) [file pone.0100537.s003.tif]

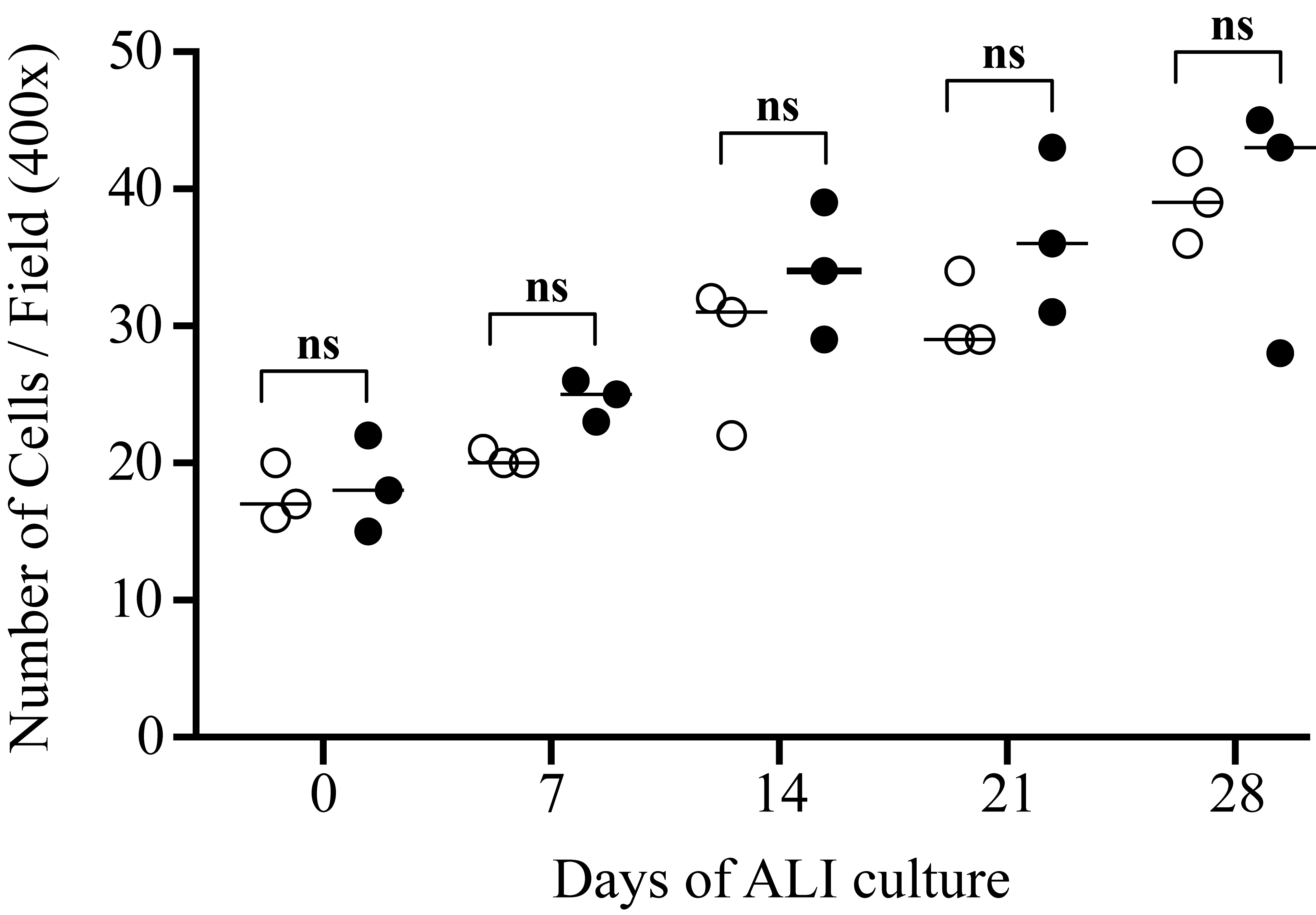

Supplement: Figure S4 — Epithelial cell counts of nasal polyp and control nasal mucosa ALI cultures at the different days analyzed. Cell counts from different nasal polyp (filled circles, n = 3) and control nasal mucosa (n = 3) ALI cultures. Results are expressed by dots (media for three replicates of each culture) and line (median of three different cultures). ns, non-significant, statistical analysis by Mann-Whitney U test (between groups). (TIF) [file pone.0100537.s004.tif]
